# 5-(6-Amino-1,3-dimethyl-2,4-dioxo-1,2,3,4-tetra­hydropyrimidin-5-yl)-1,3-dimethyl-1*H*-chromeno[2,3-*d*]pyrim­idine-2,4(3*H*,5*H*)-dione 3.5-hydrate

**DOI:** 10.1107/S1600536813013986

**Published:** 2013-05-25

**Authors:** Subhadip Roy, Subrata Das

**Affiliations:** a Department of Chemistry, National Institute of Technology-Agartala, Pin-799055, Tripura, India

## Abstract

The title compound, C_19_H_19_N_5_O_5_·3.5H_2_O, crystallizes with 3.5 mol­ecules of water in the asymmetric unit, one of which lies on a mirror plane. One of the water mol­ecules links the mol­ecules, forming centrosymmetric dimers. These dimers are then linked through further N—H⋯O and O—H⋯O hydrogen bonding, leading to the observed three-dimensional structure.

## Related literature

Many chromene derivatives occur in natural products, see: Hatakeyama *et al.* (1988 ▶). For the biological activity of functionalized chromenes, see: Brooks (1998 ▶); Valenti *et al.* (1993 ▶); Tang *et al.* (2007 ▶). For the use of 6-amino-uracil derivatives as precursors in the synthesis of biologically significant fused uracils, see: Shaw (1996 ▶). The fusion of a chromene unit to the uracil ring is found to increase the biological activity, see: Sabry *et al.* (2011 ▶). 
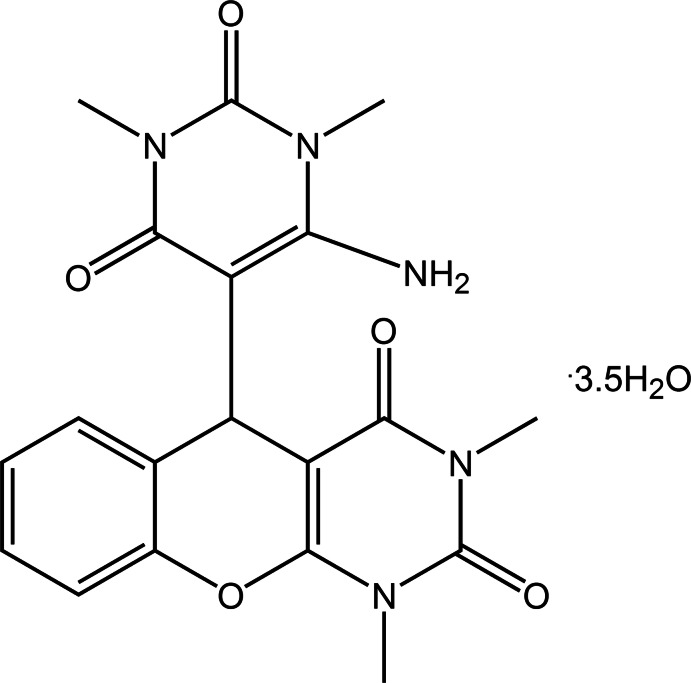


## Experimental

### 

#### Crystal data

C_19_H_19_N_5_O_5_·3.5H_2_O
*M* *_r_* = 460.45
Monoclinic, 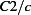

*a* = 29.993 (4) Å
*b* = 7.9105 (6) Å
*c* = 21.458 (3) Å
β = 119.860 (16)°
*V* = 4415.3 (10) Å^3^
*Z* = 8
Mo *K*α radiation
μ = 0.11 mm^−1^
*T* = 298 K
0.32 × 0.12 × 0.06 mm

#### Data collection

Oxford Diffraction Xcalibur (Eos, Gemini) diffractometer
Absorption correction: multi-scan (*CrysAlis PRO*; Oxford Diffraction, 2007 ▶) *T*_min_ = 0.93, *T*_max_ = 1.00
9327 measured reflections
4554 independent reflections
2538 reflections with *I* > 2σ(*I*)
*R*_int_ = 0.069

#### Refinement

*R*[*F* ^2^ > 2σ(*F* ^2^)] = 0.060
*wR*(*F* ^2^) = 0.137
*S* = 0.98
4554 reflections
308 parameters
H-atom parameters constrained
Δρ_max_ = 0.24 e Å^−3^
Δρ_min_ = −0.25 e Å^−3^

### 

Data collection: *CrysAlis PRO* (Oxford Diffraction, 2007 ▶); cell refinement: *CrysAlis PRO*; data reduction: *CrysAlis PRO*; program(s) used to solve structure: *OLEX*.*SOLVE* (Bourhis *et al.*, 2013 ▶); program(s) used to refine structure: *SHELXL2013* (Sheldrick, 2008 ▶); molecular graphics: *OLEX2* (Dolomanov *et al.*, 2009 ▶); software used to prepare material for publication: *OLEX2*.

## Supplementary Material

Crystal structure: contains datablock(s) global, I. DOI: 10.1107/S1600536813013986/go2089sup1.cif

Structure factors: contains datablock(s) I. DOI: 10.1107/S1600536813013986/go2089Isup2.hkl

Supplementary material file. DOI: 10.1107/S1600536813013986/go2089Isup3.cdx

Additional supplementary materials:  crystallographic information; 3D view; checkCIF report

## Figures and Tables

**Table 1:** Hydrogen-bond geometry (Å, °)

*D*—H⋯*A*	*D*—H	H⋯*A*	*D*⋯*A*	*D*—H⋯*A*
N6—H6*A*⋯O6*W* ^i^	0.86	2.18	3.009 (3)	161
N6—H6*B*⋯O8*W*	0.86	2.09	2.905 (3)	158
O6*W*—H6*WA*⋯O1^ii^	0.85	2.01	2.830 (3)	162
O6*W*—H6*WB*⋯O7*W*	0.85	2.00	2.835 (3)	167
O7*W*—H7*WA*⋯O3^iii^	0.84	1.94	2.781 (3)	177
O7*W*—H7*WB*⋯O2	0.85	1.93	2.773 (3)	170
O8*W*—H8*WA*⋯O9*W*	0.85	1.99	2.838 (4)	177
O8*W*—H8*WB*⋯O6*W* ^iv^	0.85	2.01	2.840 (3)	164
O9*W*—H9*W*⋯O7*W*	0.85	1.93	2.772 (3)	170

Symmetry codes: (i) 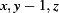; (ii) 
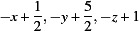
; (iii) 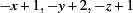; (iv) 
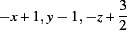
.

**Fig. 1.**
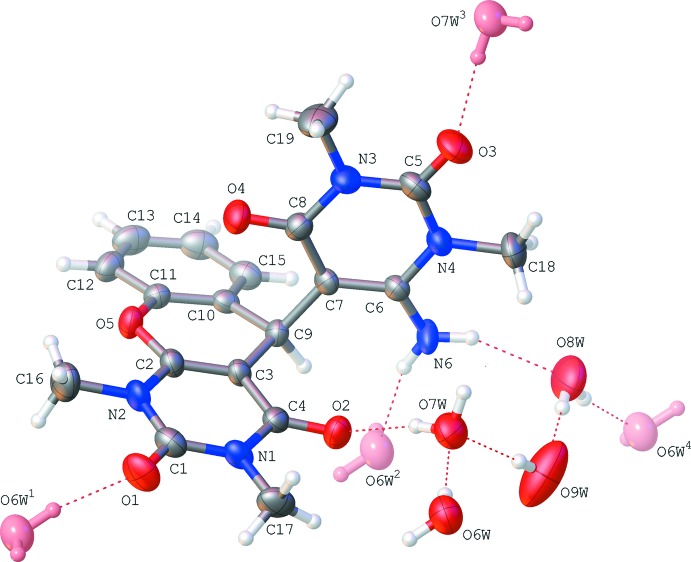
An overview of the title compound, showing the atom-numbering scheme. Symmetry-generated water molecules are shown in pink. The displacement ellipsoids are shown at the 50% probability level.

**Fig. 2.**
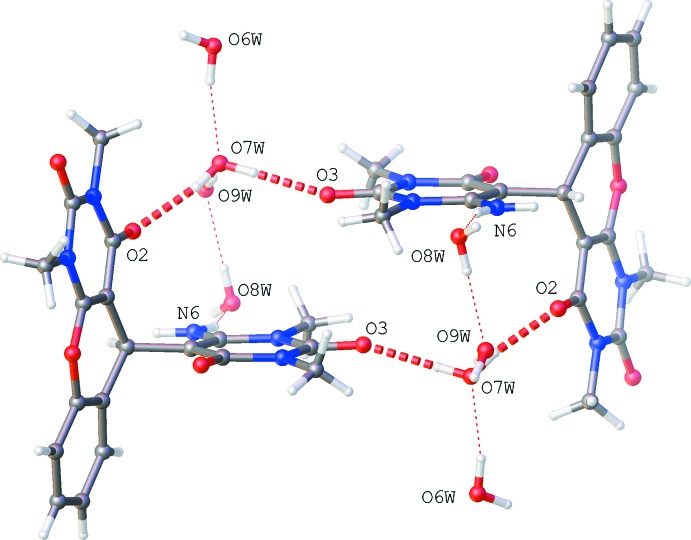
The dimers formed by the short and geometrically most optimized hydrogen bonds.

**Fig. 3.**
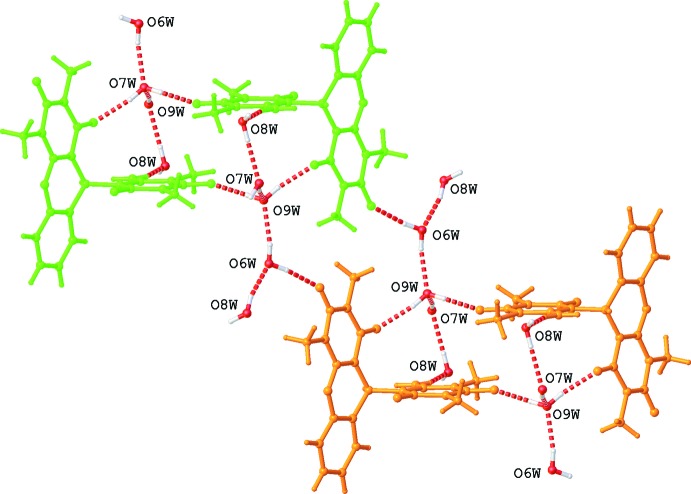
'Dimers of dimers' – the basic building blocks of the layers.

**Fig. 4.**
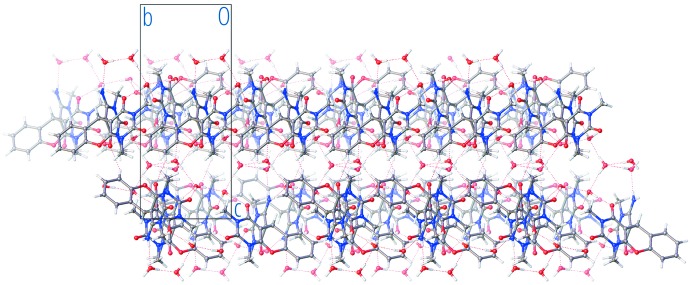
The layers as seen along the a axis.

**Fig. 5.**
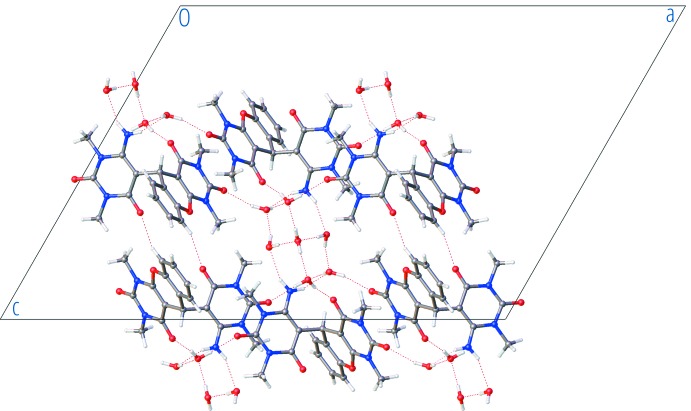
The same arrangement as in Fig. 4, but rotated by 900 and now seen along the b axis.

## supplementary crystallographic information

### Comment

Functionlized chromenes are of great interest as they have shown to possess antimicrobial, antitumoral, spasmolytic, anticoagulant and antianaphylactic characteristics (Brooks, 1998; Valenti *et al.*, 1993; Tang *et al.*, 2007). Many chromene derivatives also occur in various natural products (Hatakeyama *et al.*, 1988). 6-Amino-uracil derivatives belong to nitrogen-containing heterocycles of pyrimidine family and are used as key precursors for the synthesis of numerous biologically significant fused uracils (Shaw, 1996). The fusion of chromene unit to uracil ring is found to increase biological activity (Sabry *et al.*, 2011). In this context, the synthesis and crystal structure of the title compound are reported.

The molecules are held together through a tightly woven intermolecular hydrogen bonding network, utilizing the three and a half water molecules to establish the three-dimensional structure. The two short hydrogen bonds (D···A = 2.773 (3) and 2.781 (3) Å) show D—H···A angles of 170° and 177° respectively, which are close to the ideal 180°, yield to the formation of 'dimers' (Fig. 2), which are further strongly connected to another dimer (Fig. 3), eventually leading to layers utilizing the many hydrogen bonding interactions available to the system. Finally, these layers are held together by relatively weak C—H···O and N—H···O interactions (Fig. 4 & Fig. 5) so producing a complex three-dimensional structure.

### Experimental

Distilled water (30 ml) was added to 6-amino-1,3-dimethyluracil (1 mmol) in a 100 ml round-bottomed flask and the mixture was stirred at room temperature until all the 6-amino- 1,3-dimethyluracil had dissolved. Salicylaldehyde (0.5 mmol) was added drop wise to the 6- amino-1,3-dimethyluracil solution with constant stirring and then after 4 h the product appeared as brown precipitate and stirring was continued for further 3 h so that all of the reactants were converted into product. The brown precipitate was filtered and recrystallized from distilled ethanol to yield white transparent crystals suitable for single-crystal X-ray diffraction (yield 93%; m.p. 520–522 K).

### Refinement

The structure was solved using the charge flipping method available from olex.solve (Bourhis *et al.*, 2013) and was refined using the least squares refinement on F^2^ available from *SHELXL2013* (Sheldrick, 2008). The solution and refinement process for this structure was unremarkable and all refinement details can be inferred from the cif file itself.

### Crystal data

**Table:**

C_19_H_19_N_5_O_5_·3.5H_2_O	*Z* = 8
*M**_r_* = 460.45	*F*(000) = 1944
Monoclinic, *C*2/*c*	*D*_x_ = 1.385 Mg m^−^^3^
*a* = 29.993 (4) Å	Mo *K*α radiation, λ = 0.71073 Å
*b* = 7.9105 (6) Å	µ = 0.11 mm^−^^1^
*c* = 21.458 (3) Å	*T* = 298 K
β = 119.860 (16)°	Block, clear colourless
*V* = 4415.3 (10) Å^3^	0.32 × 0.12 × 0.06 mm

### Data collection

**Table:**

Oxford Diffraction Xcalibur (Eos, Gemini) diffractometer	4554 independent reflections
Radiation source: Enhance (Mo) X-ray Source	2538 reflections with *I* > 2σ(*I*)
Graphite monochromator	*R*_int_ = 0.069
Detector resolution: 16.1511 pixels mm^-1^	θ_max_ = 26.5°, θ_min_ = 2.8°
ω scans	*h* = −37→36
Absorption correction: multi-scan (*CrysAlis PRO*; Oxford Diffraction, 2007)	*k* = −9→5
*T*_min_ = 0.93, *T*_max_ = 1.00	*l* = −26→26
9327 measured reflections	

### Refinement

**Table:**

Refinement on *F*^2^	H-atom parameters constrained
Least-squares matrix: full	*w* = 1/[σ^2^(*F*_o_^2^) + (0.037*P*)^2^] where *P* = (*F*_o_^2^ + 2*F*_c_^2^)/3
*R*[*F*^2^ > 2σ(*F*^2^)] = 0.060	(Δ/σ)_max_ = 0.001
*wR*(*F*^2^) = 0.137	Δρ_max_ = 0.24 e Å^−^^3^
*S* = 0.98	Δρ_min_ = −0.25 e Å^−^^3^
4554 reflections	Extinction correction: *SHELXL2013* (Sheldrick, 2008), Fc^*^=kFc[1+0.001xFc^2^λ^3^/sin(2θ)]^-1/4^
308 parameters	Extinction coefficient: 0.0029 (2)
0 restraints	

### Special details

**Table:**

Geometry. All e.s.d.'s (except the e.s.d. in the dihedral angle between two l.s. planes) are estimated using the full covariance matrix. The cell e.s.d.'s are taken into account individually in the estimation of e.s.d.'s in distances, angles and torsion angles; correlations between e.s.d.'s in cell parameters are only used when they are defined by crystal symmetry. An approximate (isotropic) treatment of cell e.s.d.'s is used for estimating e.s.d.'s involving l.s. planes.

### Fractional atomic coordinates and isotropic or equivalent isotropic displacement parameters (Å^2^)

**Table:**

	*x*	*y*	*z*	*U*_iso_*/*U*_eq_	
O1	0.20391 (8)	1.0426 (2)	0.41093 (10)	0.0553 (6)	
O2	0.36262 (7)	0.8244 (2)	0.56168 (9)	0.0444 (5)	
O3	0.51160 (8)	0.8412 (3)	0.44642 (10)	0.0579 (6)	
O4	0.34405 (7)	0.6704 (2)	0.34016 (8)	0.0442 (5)	
O5	0.24740 (6)	0.5527 (2)	0.34356 (8)	0.0378 (5)	
N1	0.28226 (8)	0.9305 (3)	0.48757 (10)	0.0350 (5)	
N2	0.22732 (8)	0.8001 (3)	0.37727 (10)	0.0374 (6)	
N3	0.42861 (9)	0.7489 (3)	0.39256 (11)	0.0399 (6)	
N4	0.48358 (8)	0.7140 (3)	0.51612 (10)	0.0359 (6)	
N6	0.45814 (8)	0.5830 (3)	0.58939 (10)	0.0469 (7)	
H6A	0.4358	0.5337	0.5972	0.056*	
H6B	0.4891	0.5975	0.6240	0.056*	
C1	0.23592 (11)	0.9317 (4)	0.42411 (14)	0.0398 (7)	
C2	0.26390 (10)	0.6756 (3)	0.39378 (12)	0.0316 (6)	
C3	0.31020 (9)	0.6787 (3)	0.45432 (12)	0.0289 (6)	
C4	0.32143 (10)	0.8121 (3)	0.50451 (12)	0.0323 (6)	
C5	0.47635 (11)	0.7709 (3)	0.45087 (14)	0.0394 (7)	
C6	0.44477 (10)	0.6371 (3)	0.52362 (12)	0.0307 (6)	
C7	0.39607 (9)	0.6201 (3)	0.46424 (11)	0.0284 (6)	
C8	0.38635 (11)	0.6784 (3)	0.39612 (12)	0.0338 (6)	
C9	0.35107 (9)	0.5470 (3)	0.46939 (11)	0.0297 (6)	
H9	0.3645	0.5081	0.5189	0.036*	
C10	0.32640 (10)	0.3965 (3)	0.42059 (12)	0.0294 (6)	
C11	0.27754 (10)	0.4047 (3)	0.36074 (12)	0.0307 (6)	
C12	0.25456 (11)	0.2707 (3)	0.31460 (13)	0.0418 (7)	
H12	0.2217	0.2811	0.2751	0.050*	
C13	0.28154 (13)	0.1200 (4)	0.32827 (14)	0.0484 (8)	
H13	0.2669	0.0284	0.2975	0.058*	
C14	0.33003 (12)	0.1059 (4)	0.38743 (15)	0.0466 (8)	
H14	0.3480	0.0047	0.3966	0.056*	
C15	0.35207 (11)	0.2422 (3)	0.43328 (13)	0.0384 (7)	
H15	0.3846	0.2306	0.4733	0.046*	
C16	0.17779 (11)	0.7988 (4)	0.30914 (13)	0.0573 (9)	
H16A	0.1795	0.7193	0.2766	0.086*	
H16B	0.1709	0.9096	0.2881	0.086*	
H16C	0.1508	0.7667	0.3184	0.086*	
C17	0.29347 (11)	1.0725 (3)	0.53773 (14)	0.0498 (8)	
H17A	0.3180	1.0370	0.5856	0.075*	
H17B	0.2623	1.1077	0.5363	0.075*	
H17C	0.3074	1.1651	0.5240	0.075*	
C18	0.53408 (11)	0.7462 (4)	0.57937 (14)	0.0517 (8)	
H18A	0.5296	0.8003	0.6159	0.078*	
H18B	0.5538	0.8184	0.5662	0.078*	
H18C	0.5519	0.6410	0.5974	0.078*	
C19	0.41982 (13)	0.8013 (4)	0.32181 (13)	0.0627 (10)	
H19A	0.3910	0.7404	0.2851	0.094*	
H19B	0.4499	0.7775	0.3183	0.094*	
H19C	0.4128	0.9204	0.3157	0.094*	
O6W	0.40247 (8)	1.3900 (3)	0.65089 (12)	0.0588 (6)	
H6WA	0.3707	1.3937	0.6386	0.088*	
H6WB	0.4104	1.2891	0.6470	0.088*	
O7W	0.43622 (9)	1.0753 (3)	0.62611 (10)	0.0552 (6)	
H7WA	0.4531	1.1006	0.6055	0.083*	
H7WB	0.4155	0.9949	0.6036	0.083*	
O8W	0.54970 (10)	0.6130 (4)	0.72984 (11)	0.0811 (8)	
H8WA	0.5350	0.6963	0.7373	0.122*	
H8WB	0.5579	0.5401	0.7631	0.122*	
O9W	0.5000	0.8957 (5)	0.7500	0.1228 (18)	
H9W	0.4827	0.9612	0.7146	0.184*	

### Atomic displacement parameters (Å^2^)

**Table:**

	*U*^11^	*U*^22^	*U*^33^	*U*^12^	*U*^13^	*U*^23^
O1	0.0445 (13)	0.0462 (12)	0.0788 (14)	0.0148 (11)	0.0333 (12)	0.0016 (11)
O2	0.0366 (12)	0.0490 (12)	0.0398 (10)	−0.0016 (10)	0.0131 (9)	−0.0123 (9)
O3	0.0455 (14)	0.0749 (15)	0.0645 (13)	−0.0121 (12)	0.0357 (11)	0.0036 (11)
O4	0.0348 (12)	0.0556 (12)	0.0329 (9)	−0.0022 (10)	0.0098 (9)	0.0056 (9)
O5	0.0291 (10)	0.0419 (11)	0.0321 (9)	−0.0001 (9)	0.0075 (8)	−0.0069 (8)
N1	0.0314 (13)	0.0298 (12)	0.0480 (12)	0.0017 (11)	0.0229 (11)	−0.0050 (10)
N2	0.0227 (12)	0.0441 (14)	0.0399 (12)	0.0061 (11)	0.0114 (10)	−0.0007 (11)
N3	0.0388 (15)	0.0482 (14)	0.0377 (12)	−0.0051 (12)	0.0229 (11)	0.0031 (11)
N4	0.0251 (13)	0.0444 (13)	0.0371 (11)	0.0000 (11)	0.0146 (10)	0.0003 (10)
N6	0.0248 (13)	0.0726 (17)	0.0309 (11)	−0.0112 (13)	0.0044 (10)	0.0060 (11)
C1	0.0354 (16)	0.0398 (16)	0.0509 (16)	0.0040 (14)	0.0266 (14)	0.0040 (14)
C2	0.0241 (14)	0.0342 (14)	0.0349 (13)	−0.0023 (12)	0.0134 (12)	−0.0023 (11)
C3	0.0232 (14)	0.0298 (14)	0.0326 (12)	−0.0032 (11)	0.0131 (11)	−0.0041 (11)
C4	0.0302 (15)	0.0366 (15)	0.0333 (13)	−0.0059 (13)	0.0181 (12)	−0.0033 (11)
C5	0.0329 (17)	0.0404 (16)	0.0521 (17)	0.0039 (14)	0.0268 (14)	0.0025 (14)
C6	0.0263 (14)	0.0345 (14)	0.0327 (13)	0.0023 (12)	0.0158 (11)	−0.0005 (12)
C7	0.0222 (14)	0.0303 (14)	0.0312 (12)	0.0054 (11)	0.0121 (11)	0.0025 (11)
C8	0.0340 (16)	0.0348 (15)	0.0313 (13)	0.0019 (13)	0.0153 (12)	−0.0010 (11)
C9	0.0246 (14)	0.0342 (14)	0.0260 (12)	−0.0001 (12)	0.0094 (11)	−0.0015 (11)
C10	0.0302 (15)	0.0307 (14)	0.0333 (13)	−0.0038 (12)	0.0204 (12)	−0.0032 (11)
C11	0.0312 (15)	0.0337 (14)	0.0284 (12)	0.0008 (12)	0.0157 (11)	−0.0024 (11)
C12	0.0442 (18)	0.0456 (17)	0.0338 (14)	−0.0154 (15)	0.0182 (13)	−0.0104 (13)
C13	0.065 (2)	0.0394 (17)	0.0512 (17)	−0.0101 (17)	0.0370 (17)	−0.0134 (14)
C14	0.060 (2)	0.0339 (16)	0.0600 (18)	−0.0009 (15)	0.0404 (17)	−0.0045 (14)
C15	0.0395 (17)	0.0361 (15)	0.0443 (15)	0.0054 (14)	0.0243 (13)	0.0037 (13)
C16	0.0357 (19)	0.069 (2)	0.0505 (17)	0.0128 (17)	0.0093 (15)	−0.0031 (16)
C17	0.057 (2)	0.0424 (17)	0.0625 (18)	−0.0016 (16)	0.0394 (17)	−0.0147 (15)
C18	0.0275 (16)	0.069 (2)	0.0514 (17)	−0.0142 (16)	0.0140 (14)	−0.0073 (16)
C19	0.073 (3)	0.080 (2)	0.0452 (16)	−0.018 (2)	0.0366 (17)	0.0045 (17)
O6W	0.0444 (14)	0.0660 (15)	0.0617 (13)	−0.0016 (12)	0.0231 (12)	−0.0009 (12)
O7W	0.0469 (15)	0.0627 (15)	0.0571 (12)	−0.0127 (12)	0.0267 (11)	−0.0025 (11)
O8W	0.0576 (17)	0.114 (2)	0.0506 (13)	0.0053 (16)	0.0110 (12)	0.0133 (14)
O9W	0.157 (4)	0.076 (3)	0.060 (2)	0.000	−0.002 (2)	0.000

### Geometric parameters (Å, º)

**Table:**

O1—C1	1.225 (3)	C10—C11	1.388 (3)
O2—C4	1.238 (3)	C10—C15	1.396 (3)
O3—C5	1.240 (3)	C11—C12	1.378 (3)
O4—C8	1.240 (3)	C12—H12	0.9300
O5—C2	1.349 (3)	C12—C13	1.388 (4)
O5—C11	1.411 (3)	C13—H13	0.9300
N1—C1	1.379 (3)	C13—C14	1.379 (4)
N1—C4	1.401 (3)	C14—H14	0.9300
N1—C17	1.474 (3)	C14—C15	1.385 (3)
N2—C1	1.379 (3)	C15—H15	0.9300
N2—C2	1.383 (3)	C16—H16A	0.9600
N2—C16	1.477 (3)	C16—H16B	0.9600
N3—C5	1.365 (3)	C16—H16C	0.9600
N3—C8	1.421 (3)	C17—H17A	0.9600
N3—C19	1.465 (3)	C17—H17B	0.9600
N4—C5	1.381 (3)	C17—H17C	0.9600
N4—C6	1.392 (3)	C18—H18A	0.9600
N4—C18	1.468 (3)	C18—H18B	0.9600
N6—H6A	0.8600	C18—H18C	0.9600
N6—H6B	0.8600	C19—H19A	0.9600
N6—C6	1.331 (3)	C19—H19B	0.9600
C2—C3	1.349 (3)	C19—H19C	0.9600
C3—C4	1.424 (3)	O6W—H6WA	0.8524
C3—C9	1.516 (3)	O6W—H6WB	0.8486
C6—C7	1.386 (3)	O7W—H7WA	0.8449
C7—C8	1.418 (3)	O7W—H7WB	0.8516
C7—C9	1.522 (3)	O8W—H8WA	0.8524
C9—H9	0.9800	O8W—H8WB	0.8519
C9—C10	1.513 (3)	O9W—H9W	0.8500
			
C2—O5—C11	117.17 (18)	C11—C10—C9	122.1 (2)
C1—N1—C4	124.3 (2)	C11—C10—C15	116.6 (2)
C1—N1—C17	117.8 (2)	C15—C10—C9	121.4 (2)
C4—N1—C17	117.6 (2)	C10—C11—O5	121.6 (2)
C1—N2—C2	121.0 (2)	C12—C11—O5	115.1 (2)
C1—N2—C16	117.2 (2)	C12—C11—C10	123.3 (2)
C2—N2—C16	121.8 (2)	C11—C12—H12	120.7
C5—N3—C8	123.9 (2)	C11—C12—C13	118.6 (3)
C5—N3—C19	118.6 (2)	C13—C12—H12	120.7
C8—N3—C19	117.5 (2)	C12—C13—H13	120.0
C5—N4—C6	122.8 (2)	C14—C13—C12	120.0 (3)
C5—N4—C18	116.7 (2)	C14—C13—H13	120.0
C6—N4—C18	120.4 (2)	C13—C14—H14	119.9
H6A—N6—H6B	120.0	C13—C14—C15	120.2 (3)
C6—N6—H6A	120.0	C15—C14—H14	119.9
C6—N6—H6B	120.0	C10—C15—H15	119.3
O1—C1—N1	121.4 (3)	C14—C15—C10	121.3 (3)
O1—C1—N2	122.1 (3)	C14—C15—H15	119.3
N1—C1—N2	116.4 (2)	N2—C16—H16A	109.5
O5—C2—N2	112.3 (2)	N2—C16—H16B	109.5
O5—C2—C3	125.1 (2)	N2—C16—H16C	109.5
C3—C2—N2	122.6 (2)	H16A—C16—H16B	109.5
C2—C3—C4	119.0 (2)	H16A—C16—H16C	109.5
C2—C3—C9	121.9 (2)	H16B—C16—H16C	109.5
C4—C3—C9	119.1 (2)	N1—C17—H17A	109.5
O2—C4—N1	119.9 (2)	N1—C17—H17B	109.5
O2—C4—C3	123.5 (2)	N1—C17—H17C	109.5
N1—C4—C3	116.6 (2)	H17A—C17—H17B	109.5
O3—C5—N3	122.2 (2)	H17A—C17—H17C	109.5
O3—C5—N4	121.0 (3)	H17B—C17—H17C	109.5
N3—C5—N4	116.8 (2)	N4—C18—H18A	109.5
N6—C6—N4	115.8 (2)	N4—C18—H18B	109.5
N6—C6—C7	124.5 (2)	N4—C18—H18C	109.5
C7—C6—N4	119.7 (2)	H18A—C18—H18B	109.5
C6—C7—C8	119.9 (2)	H18A—C18—H18C	109.5
C6—C7—C9	122.5 (2)	H18B—C18—H18C	109.5
C8—C7—C9	117.6 (2)	N3—C19—H19A	109.5
O4—C8—N3	118.5 (2)	N3—C19—H19B	109.5
O4—C8—C7	124.6 (3)	N3—C19—H19C	109.5
C7—C8—N3	116.9 (2)	H19A—C19—H19B	109.5
C3—C9—C7	112.1 (2)	H19A—C19—H19C	109.5
C3—C9—H9	107.4	H19B—C19—H19C	109.5
C7—C9—H9	107.4	H6WA—O6W—H6WB	109.1
C10—C9—C3	109.15 (19)	H7WA—O7W—H7WB	109.8
C10—C9—C7	113.24 (19)	H8WA—O8W—H8WB	109.1
C10—C9—H9	107.4		
			
O5—C2—C3—C4	−176.7 (2)	C7—C9—C10—C15	68.9 (3)
O5—C2—C3—C9	5.0 (4)	C8—N3—C5—O3	−176.0 (2)
O5—C11—C12—C13	−179.4 (2)	C8—N3—C5—N4	2.7 (4)
N2—C2—C3—C4	2.1 (4)	C8—C7—C9—C3	−65.0 (3)
N2—C2—C3—C9	−176.2 (2)	C8—C7—C9—C10	59.0 (3)
N4—C6—C7—C8	1.1 (4)	C9—C3—C4—O2	−2.4 (4)
N4—C6—C7—C9	−176.3 (2)	C9—C3—C4—N1	179.7 (2)
N6—C6—C7—C8	−178.3 (2)	C9—C7—C8—O4	−1.7 (4)
N6—C6—C7—C9	4.3 (4)	C9—C7—C8—N3	179.0 (2)
C1—N1—C4—O2	177.3 (2)	C9—C10—C11—O5	−2.3 (4)
C1—N1—C4—C3	−4.7 (4)	C9—C10—C11—C12	178.7 (2)
C1—N2—C2—O5	176.3 (2)	C9—C10—C15—C14	−178.2 (2)
C1—N2—C2—C3	−2.6 (4)	C10—C11—C12—C13	−0.2 (4)
C2—O5—C11—C10	−11.2 (3)	C11—O5—C2—N2	−168.9 (2)
C2—O5—C11—C12	167.9 (2)	C11—O5—C2—C3	10.0 (4)
C2—N2—C1—O1	−179.3 (2)	C11—C10—C15—C14	1.2 (4)
C2—N2—C1—N1	−0.5 (4)	C11—C12—C13—C14	0.7 (4)
C2—C3—C4—O2	179.3 (2)	C12—C13—C14—C15	−0.2 (4)
C2—C3—C4—N1	1.4 (4)	C13—C14—C15—C10	−0.8 (4)
C2—C3—C9—C7	109.6 (3)	C15—C10—C11—O5	178.3 (2)
C2—C3—C9—C10	−16.7 (3)	C15—C10—C11—C12	−0.7 (4)
C3—C9—C10—C11	15.2 (3)	C16—N2—C1—O1	0.9 (4)
C3—C9—C10—C15	−165.5 (2)	C16—N2—C1—N1	179.7 (2)
C4—N1—C1—O1	−176.9 (2)	C16—N2—C2—O5	−4.0 (3)
C4—N1—C1—N2	4.3 (4)	C16—N2—C2—C3	177.1 (2)
C4—C3—C9—C7	−68.7 (3)	C17—N1—C1—O1	−3.3 (4)
C4—C3—C9—C10	165.0 (2)	C17—N1—C1—N2	177.9 (2)
C5—N3—C8—O4	177.1 (2)	C17—N1—C4—O2	3.7 (4)
C5—N3—C8—C7	−3.5 (4)	C17—N1—C4—C3	−178.4 (2)
C5—N4—C6—N6	177.5 (2)	C18—N4—C5—O3	2.0 (4)
C5—N4—C6—C7	−2.0 (4)	C18—N4—C5—N3	−176.7 (2)
C6—N4—C5—O3	178.9 (3)	C18—N4—C6—N6	−5.8 (4)
C6—N4—C5—N3	0.1 (4)	C18—N4—C6—C7	174.7 (2)
C6—C7—C8—O4	−179.2 (2)	C19—N3—C5—O3	3.7 (4)
C6—C7—C8—N3	1.5 (3)	C19—N3—C5—N4	−177.6 (2)
C6—C7—C9—C3	112.4 (3)	C19—N3—C8—O4	−2.5 (4)
C6—C7—C9—C10	−123.6 (2)	C19—N3—C8—C7	176.8 (2)
C7—C9—C10—C11	−110.4 (3)		

### Hydrogen-bond geometry (Å, º)

**Table:**

*D*—H···*A*	*D*—H	H···*A*	*D*···*A*	*D*—H···*A*
N6—H6*A*···O6*W*^i^	0.86	2.18	3.009 (3)	161
N6—H6*B*···O8*W*	0.86	2.09	2.905 (3)	158
O6*W*—H6*WA*···O1^ii^	0.85	2.01	2.830 (3)	162
O6*W*—H6*WB*···O7*W*	0.85	2.00	2.835 (3)	167
O7*W*—H7*WA*···O3^iii^	0.84	1.94	2.781 (3)	177
O7*W*—H7*WB*···O2	0.85	1.93	2.773 (3)	170
O8*W*—H8*WA*···O9*W*	0.85	1.99	2.838 (4)	177
O8*W*—H8*WB*···O6*W*^iv^	0.85	2.01	2.840 (3)	164
O9*W*—H9*W*···O7*W*	0.85	1.93	2.772 (3)	170

Symmetry codes: (i) *x*, *y*−1, *z*; (ii) −*x*+1/2, −*y*+5/2, −*z*+1; (iii) −*x*+1, −*y*+2, −*z*+1; (iv) −*x*+1, *y*−1, −*z*+3/2.
